# Genome-wide identification of *CAMTA* gene family members in *Medicago truncatula* and their expression during root nodule symbiosis and hormone treatments

**DOI:** 10.3389/fpls.2015.00459

**Published:** 2015-06-19

**Authors:** Yanjun Yang, Tao Sun, Luqin Xu, Erxu Pi, Sheng Wang, Huizhong Wang, Chenjia Shen

**Affiliations:** College of Life and Environmental Sciences, Hangzhou Normal UniversityHangzhou, China

**Keywords:** calcium, *CAMTA* gene family, *Medicago truncatula*, *Sinorhizobium meliloti* infection, nodule formation

## Abstract

Calmodulin-binding transcription activators (CAMTAs) are well-characterized calmodulin-binding transcription factors in the plant kingdom. Previous work shows that CAMTAs play important roles in various biological processes including disease resistance, herbivore attack response, and abiotic stress tolerance. However, studies that address the function of CAMTAs during the establishment of symbiosis between legumes and rhizobia are still lacking. This study undertook comprehensive identification and analysis of *CAMTA* genes using the latest updated *M. truncatula* genome. All the *MtCAMTA* genes were expressed in a tissues-specific manner and were responsive to environmental stress-related hormones. The expression profiling of *MtCAMTA* genes during the early phase of *Sinorhizobium meliloti* infection was also analyzed. Our data showed that the expression of most *MtCAMTA* genes was suppressed in roots by *S. meliloti* infection. The responsiveness of *MtCAMTAs* to *S. meliloti* infection indicated that they may function as calcium-regulated transcription factors in the early nodulation signaling pathway. In addition, bioinformatics analysis showed that CAMTA binding sites existed in the promoter regions of various early rhizobial infection response genes, suggesting possible MtCAMTAs-regulated downstream candidate genes during the early phase of *S. meliloti* infection. Taken together, these results provide basic information about *MtCAMTAs* in the model legume *M. truncatula*, and the involvement of MtCAMTAs in nodule organogenesis. This information furthers our understanding of MtCAMTA protein functions in *M. truncatula* and opens new avenues for continued research.

## Introduction

Ca^2+^ signals are core transducers and regulators in many adaptive and developmental plant processes (Kudla et al., 2010). Ca^2+^ signals are decoded and transmitted by several types of Ca^2+^ binding proteins that contain a highly conserved Ca^2+^-binding EF-hand motif (Kudla et al., 2010; Du et al., 2011). The three main classes of Ca^2+^ sensors in plants are calmodulins (together with calmodulin-like proteins) (CaMs/CMLs), calcium-dependent protein kinases (CDPKs), and calcineurin B-like proteins (CBLs) (DeFalco et al., 2010). Calmodulin is the best characterized Ca^2+^ binding protein whose role relies on its ability to physically bind to a large population of target proteins, including protein kinases, phosphatases, transcription factors, metabolic enzymes, ion channels, transporters, and molecular motors (Yang and Poovaiah, 2003; Bouche' et al., 2005; Du et al., 2011; Poovaiah et al., 2013).

In recent years, over 90 transcription factors have been identified as CaM-binding proteins (CBPs), including CAMTAs (also known as AtSRs), MYBs, WRKY IIDs, bZIPs, CBP60s, NACs, and MADS box proteins (Reddy et al., 2002, 2011; Popescu et al., 2007; Galon et al., 2010a). Calmodulin-binding transcription activators (CAMTAs), the well-studied CaM-binding transcription factors, exist in all multicellular organisms (Bouche' et al., 2002). CAMTAs are characterized by a CG-1 DNA binding domain at the N terminus, a TIG domain involved in non-specific DNA binding, several Ankyrin repeats responsible for mediating protein-protein interactions, a Ca^2+^-dependent CaM binding domain (CaMBD), and a varying number of IQ motifs which are Ca^2+^-independent CaM-binding motifs (Bouche' et al., 2002; Yang and Poovaiah, 2002; Finkler et al., 2007; Du et al., 2009). Ca^2+^/calmodulin binds to CAMTAs' CaM binding domain, and this binding regulates the activity of CAMTAs as transcriptional factors (Bouche' et al., 2002; Yang and Poovaiah, 2002; Choi et al., 2005; Du et al., 2009). The CAMTAs can specifically recognize and bind to (A/C/G)CGCG(T/C/G) or (A/C)CGTGT DNA *cis*-element in the promoter regions of downstream genes, which results in the regulation of gene expression (Yang and Poovaiah, 2002; Choi et al., 2005).

NtER1 from tobacco was the first member of the CAMTA family isolated in a screen for CaM-binding proteins (Yang and Poovaiah, 2000). In *Arabidopsis*, there are six *CAMTAs* (*AtCAMTA1* to *AtCAMTA6*), with expressions that are highly responsive to environmental signals such as temperature extremes, UVB, salt, and wounding, as well as hormones such as ethylene, jasmonate acid (JA), abscisic acid (ABA), salicylic acid (SA), and auxin (Reddy et al., 2000; Yang and Poovaiah, 2000, 2002; Galon et al., 2010b). Loss-of-function *CAMTA3/AtSR1* mutants showed chlorosis and autonomous lesions, and elevated resistance to pathogens (Galon et al., 2008; Du et al., 2009). These phenotypes were correlated with elevated levels of endogenous SA, suggesting that CAMTA3/AtSR1 was a negative regulator of SA-mediated defense responses (Du et al., 2009). Similarly, the mutant of a rice CAMTA member OsCBT showed significant resistance to pathogens, indicating that OsCBT might also act as a negative regulator on plant defense (Koo et al., 2009). CAMTA3 also played important roles in plant defense against insect herbivore, the regulation of glucose metabolism, and ethylene-induced senescence in *Arabidopsis* (Laluk et al., 2012; Nie et al., 2012; Qiu et al., 2012). Recently, *CAMTA1*, *CAMTA2*, and *CAMTA3* were reported to function together in suppressing SA biosynthesis and were involved in freezing tolerance by CBF transcription induction (Doherty et al., 2009; Kim et al., 2013b).

The symbiotic relationships between legume roots and rhizobia bacteria leads to the formation of unique structures called nodules, where the bacteria fix atmospheric dinitrogen into ammonia for plant use. A number of studies suggest that Ca^2+^ and calmodulin are critical players in plant responses to symbionts (Oldroyd and Downie, 2006; Ranty et al., 2006). In the process of symbiosis establishment, plant roots secrete flavonoids, which stimulate the bacterial synthesis of lipochitooligosaccharide called Nod Factors (NFs) (Denarie et al., 1996; Spaink, 2000). Perception of NFs and rhizobia lead to rapid Ca^2+^ influx and Ca^2+^ spiking in legumes and the transduction of Nod factor signal relies on Ca^2+^ signal transduction (Shaw and Long, 2003; Oldroyd and Downie, 2006; Charpentier and Oldroyd, 2013). A calcium-calmodulin-dependent protein kinase (CCaMK) was shown to be a key decoder of calcium signal and integrally involved in the early events of symbioses (Singh and Parniske, 2012). CCaMK contains three EF hands for calcium binding and a calmodulin-binding domain (Swainsbury et al., 2012). Binding of Ca^2+^ and Ca^2+^/calmodulin inhibits autophosphorylation of CCaMK and activates the protein as protein kinase (Mitra et al., 2004). In early nodulation progress, CCaMK is highly sensitive to changes in calcium levels and regulate expression of various nodule organogenesis genes by phosphorylation and activation of downstream targets as protein kinase (Lévy et al., 2004; Mitra et al., 2004; Swainsbury et al., 2012). CYCLOPS, a DNA-binding transcriptional activator, is a direct phosphorylation substrate of CCaMK (Yano et al., 2008; Singh et al., 2014). The phosphorylation of CYCLOPS activates nodule organogenesis, genes expression and is essential for symbiosis (Singh et al., 2014). Global gene expression profiles in nodules and analysis of *Medicago* cDNA libraries indicated that several CaM and CaML genes as well as genes encoding calcium and calmodulin binding proteins were found to be expressed in *Medicago* and *Lotus* nodules (Fedorova et al., 2002; Colebatch et al., 2004; Moreau et al., 2011). Six *M. truncatula* CaML proteins were reported to be transferred out of the cytoplasm into the symbiosome space, a matrix-filled space surrounding the bacteroid, and were likely candidates for mediating signal transduction and/or communication between the host plant and microbial symbiont (Liu et al., 2006).

To date, most works on *CAMTA* genes have focused on *Arabidopsis*. The expression pattern analysis of *CAMTA* genes in other plant species may provide preliminary clues on their probable biological functions. *CAMTAs* from tomato were found to be differentially expressed during fruit development and ripening processes, indicating that calcium signaling is involved in the regulation of fruit development and ripening through calcium/calmodulin/CAMTA interactions (Yang et al., 2012). Very recently, 15 *CAMTA* genes were identified in soybean (a legume that forms determinate type of nodules), and expression pattern analysis showed that they were responsive to various stresses and hormone signals (Wang et al., 2014). Although this work helped us to gain a preliminary impression of the legume CAMTAs, however, information about CAMTAs in *M. truncatula* which is a model legume that forms indeterminate type of nodules for symbiosis is still lacking. In this study, we report the identification and a comprehensive analysis of the *CAMTA* gene family in *M. truncatula*. Specifically, detailed information is provided on the gene structures, chromosomal locations, and promoter *cis*-element identification of seven *CAMTA* genes in *M. truncatula*. Tissue-specific expression patterns, responses to hormone treatment, and involvement of *MtCAMTA* genes in symbiosis were also analyzed. Compared to the previous work which emphasized *CAMTAs*' response to stress in soybean (Wang et al., 2014), this study is mainly focused on the possible role of *MtCAMTA* genes in nodulation and symbiosis, and extends the analysis to the transcriptional regulation during early interactions with rhizobium bacteria. The distinct spatio-temporal expression patterns for *M. truncatula CAMTA* genes and their differential responses to rhizobial symbiosis provide basic information about *M. truncatula CAMTA* genes and offer fundamental clues about their involvement in nodule organogenesis.

## Materials and methods

### Plant material, growth conditions, and hormone treatment

*M. truncatula* cv Jemalong A17 was used throughout the experiment. Seeds were scarified using concentrated sulfuric acid for 10 min, surface sterilized with 6.25% (v/v) hypochlorite for 5 min, and washed five times with sterile water. Seeds were germinated on 1% deionized water agar plates in the dark overnight at 30°C. After germination, seedlings were grown hydroponically in buffered nodulation medium (BNM) (Engstrom et al., 2002), and the nutrient solution was changed every 3 days. The seedlings were incubated in a growth chamber at a constant 22°C over a 16 h day and 8 h night with a photon flux density of 100 μmol m^−2^ s^−1^. For hormone treatment, 14-day-old seedlings (10 seedlings) were flood incubated in 1 μM indole-3-acetic acid (IAA) (Breakspear et al., 2014), 0.5 mM SA (Palma et al., 2013), 100 μM methyl jasmonate (Me-JA) (Zhang et al., 2012), and 100 μM ABA (Gimeno-Gilles et al., 2009), respectively. Roots of the hormone-treated and non-treated plants were collected at time intervals of 0, 1, 6, 12, and 24 h. After collection, all the samples were immediately frozen in liquid nitrogen and stored at −80°C for RNA extraction. Five biological repetitions were used in this experiment.

### Identification of putative *MtCAMTA* genes

Six previously-reported amino acid sequences of *Arabidopsis CAMTAs* were used as query probes to search the phytozome v10 database (http://phytozome.jgi.doe.gov/pz/portal.html) using the BLAST program. The *e*-value acceptable in the BLAST analysis for CAMTA member identification was “−3.” The hidden Markov model (HMM) profiles of the CAMTA protein family (Pfam 03859: CG-1 DNA-binding domain; Pfam 01833: TIG domain; Pfam 12796: ankyrin repeats; Pfam 00612: IQ motifs) were employed to identify MtCAMTA proteins from the hits. All the obtained sequences were sorted as unique sequences for further protein domain search in the Pfam database (http://pfam.xfam.org/). Sequences lacking one or more conserved CAMTA domains were discarded. After correcting from the resulting hits, the remaining non-redundant sequences with the highest similarity to the query sequences were retained as putative *MtCAMTA* genes.

### Gene structure and phylogenetic relationship analysis

Genomic, transcript, CDS, and amino acid sequences of *MtCAMTA* members were downloaded from Phytozome v10 database (http://phytozome.jgi.doe.gov/pz/portal.html). The schematic structures of *MtCAMTA* members, based on exon/intron data, were analyzed at the Gene Structure Display Server (http://gsds2.cbi.pku.edu.cn/index.php) (Hu et al., 2014). Protein domain structures were analyzed in the Pfam database and a schematic diagram was constructed using Domain Illustrator software (http://dog.biocuckoo.org/) (Ren et al., 2009). The calmodulin binding domain was analyzed in the Calmodulin Target Database (http://www.calmodulin.org/calmodulin-target-database/calmodulin_function/). The Compute pI/Mw tool of ExPASy (http://web.expasy.org/compute_pi/) was used to predict molecular weights and isoelectric points of the deduced MtCAMTA proteins. A phylogenetic tree was constructed using MEGA 6.0 software (http://www.megasoftware.net/mega.php) employing the neighbor-joining (NJ) method with 1000 bootstrap replicates.

### Analysis of *cis*-elements

To investigate *cis*-elements in the promoter sequences of the *MtCAMTA* genes, 2 kb of genomic DNA sequences upstream of the initiation codon (ATG) were obtained from the phytozome v10 database. The putative *cis*-elements of *MtCAMTAs* were predicted using the website of plant *cis*-acting regulatory DNA elements (PLACE) (http://www.dna.affrc.go.jp/PLACE/signalscan.html).

### Bacterial strains and rhizobia infection

Seven-day-old seedlings were transferred to nitrogen-free BNM medium for an additional 7 days before inoculation with *S. meliloti* strain 1021 (from ATCC database, ATCC® Number: 51124). The strain was grown in liquid LBMC medium (Cowie et al., 2006) containing 10 g/L tryptone, 5 g/L yeast extract, 10 g/L NaCl, 2.6 mM MgSO_4_, 2.6 mM CaCl_2_, and supplemented with 200 μg/mL streptomycin at 28°C for 48 h. For the inoculations, bacteria were pelleted, washed three times with sterile distilled water and finally diluted in nitrogen-free BNM medium to OD_600_ of 0.1. For plant inoculation, each seedling was placed in a single 25 ml tube containing the inoculum. For control, the seedlings were treated with nitrogen-free BNM medium only. Roots of the non-inoculated and *S. meliloti*-inoculated plants were collected at time intervals of 0, 1, 6, 12, 24, 28, and 72 h. After collection, all the samples were immediately frozen in liquid nitrogen and stored at −80°C for RNA extraction. Five biological repetitions were used in this experiment. The nodules were harvested at 3 weeks after inoculation and were used for RNA extraction.

### RNA isolation and quantitative RT-PCR analysis

Total RNA from samples was extracted using RNeasy plant mini kits (Qiagen, Hilden, Germany) following the manufacturer's protocol, and digested with DNase I to remove genomic DNA contamination. First strand cDNA was prepared using M-MLV reverse transcriptase (CoWin Biotech, Beijing, China) according to the manufacturer's instructions. The gene-specific primers sequences of qRT-PCR were designed using Primer Premier 5 software (PREMIER Biosoft International, Palo Alto, CA) and are shown in Table S1. Triplicate quantitative assays were performed on 1 μl of each cDNA dilution using SYBR Green Master Mix (CoWin Biotech, Beijing, China) with a MyiQ Single Color Real-time PCR system (Bio-Rad, Hercules, CA, USA), according to the manufacturer's protocol. The procedures for PCR were as follows: 95°C for 10 min; 40 cycles of 95°C for 15 s, and 60°C for 60 s. The expression level of the *MtActin* (*MTR_2g008050*) gene was used as the endogenous control to calculate relative fold differences based on comparative cycle threshold (2^−ΔΔCt^) values. All the experiments were repeated five times. For data statistical analysis, a given fold change value (two-fold) in the expression levels is used to clarify the significant differences among the control and the treatments.

## Results

### Genome-wide identification of *CAMTA* genes in *M. truncatula*

**Figure 1:**
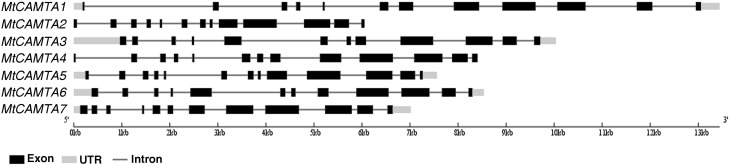
**Gene structures of** ***MtCAMTA*** **genes**. The exon-intron structures of *MtCAMTA* genes were determined by comparing the coding sequences and the corresponding genomic sequences using the Gene Structure Display Server (GSDS, http://gsds.cbi.pku.edu.cn/). The black box indicates exons; the gray box indicates 5′- and 3′-untranslated regions; the black line indicates introns.

A total of seven putative *MtCAMTA* genes were identified at the phytozome website (http://phytozome.jgi.doe.gov/pz/portal.html). They were named *MtCAMTA1* to *MtCAMTA7* according to their positions on the *M. truncatula* chromosomes. All of the information on these seven genes, such as gene names, locus IDs, ORF lengths, numbers of introns, locations on chromosome and basic information about deduced polypeptides are provided in Table 1. The predicted MtCAMTA proteins contain 914 (MtCAMTA2) to 1086 (MtCAMTA7) amino-acid residues that are similar to CAMTA members from other organisms (Yang and Poovaiah, 2002; Koo et al., 2009; Yang et al., 2012; Wang et al., 2014). The predicted molecular masses and isoelectric point (pI) range from 103.66 kDa (MtCAMTA2) to 123.62 kDa (MtCAMTA7) and 5.51 (MtCAMTA3) to 7.64 (MtCAMTA5), respectively. Gene structure analysis showed that intron numbers contained in *MtCAMTAs* range from 11 to 12 (Table 1, Figure 1).

**Table 1:** **CAMTA gene family in** ***Medicago truncatula***.

**Gene**	**Locus ID**	**ORF length (bp)**	**No. of introns**	**Chr. no**.	**Chr. location**	**Deduced polypeptide**		
						**Length (aa)**	**Mol wt (kDa)**	**pI**
*MtCAMTA1*	Medtr2g008840	3693	11	2	1646546–1659991	1040	116.77	5.63
*MtCAMTA2*	Medtr2g034650	2749	12	2	13256129–13262185	914	103.66	6.55
*MtCAMTA3*	Medtr3g085050	3405	11	3	38424643–38434678	958	107.84	5.51
*MtCAMTA4*	Medtr4g094215	3093	12	4	37451958–37460364	1030	115.97	5.73
*MtCAMTA5*	Medtr4g121840	2763	12	4	50265218–50272778	924	105.30	7.64
*MtCAMTA6*	Medtr8g080800	3123	11	8	34780716–34789253	961	107.60	5.59
*MtCAMTA7*	Medtr8g090205	3771	11	8	37838861–37845878	1086	123.62	5.78

### Phylogenetic and structural analysis of MtCAMTA proteins in *M. truncatula*

**Figure 2:**
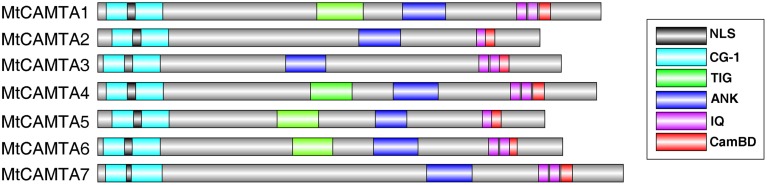
**Schematic diagram of the protein domain structures of MtCAMTAs**. Analysis of functional conserved domains were performed in the Pfam database (http://pfam.janelia.org/). Nuclear localization signals (NLS) were searched by Motif scan (http://myhits.isb-sib.ch/cgi-bin/motif_scan). CaM-binding domains (CaMBD) were analyzed in the Calmodulin Target Database (http://calcium.uhnres.utoronto.ca/ctdb/ctdb/). The domain structures of MtCAMTAs were drawn using Domain Graph software (http://dog.biocuckoo.org/). NLS, Nuclear localization signals; CG-1, CG-1 DNA binding domain; TIG, TIG domain involved in non-specific DNA binding, ANK, Ankyrin repeats responsible for mediating protein-protein interactions; IQ, Ca^2+^-independent CaM-binding IQ motifs, CamBD, Ca^2+^-dependent CaM binding domain.

The domain structures of MtCAMTA proteins were analyzed in the Pfam database (Punta et al., 2012). MtCAMTA1, 4, 5, and 6 were predicted to contain all the conserved domains of a typical CAMTA protein, including a CG-1 DNA-binding domain (Pfam 03859), a TIG domain involved in non-specific DNA binding (Pfam 01833), several ankyrin repeats (Pfam 12796), one or two IQ motifs which are Ca^2+^-independent CaM-binding motifs (Pfam 00612), and a Ca^2+^ dependent calmodulin binding domain (Figure 2). Meanwhile, MtCAMTA 2, 3, and 7 contained all of the conserved domains except for the TIG domain, which is involved in non-specific DNA binding (Figure 2). All of the MtCAMTA proteins were predicted to contain a nuclear localization signal (NLS) in the N-terminus of the protein, consistent with their function in the nucleus as transcription factors (Figure 2).

**Figure 3:**
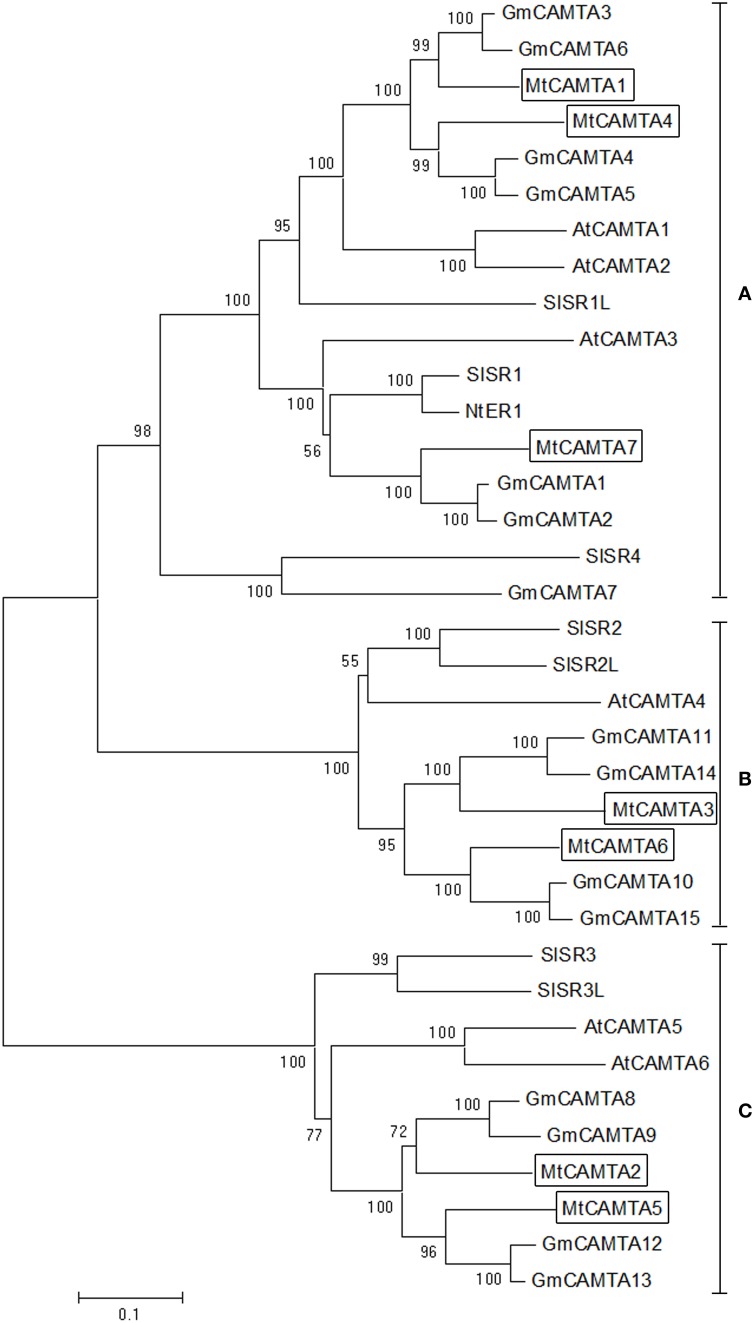
**Phylogenetic tree of** ***CAMTA*** **homologs from dicot plants**. Phylogenetic trees of *CAMTAs* from five dicot plants, including *M. truncatula*, *Arabidopsis*, soybean, tobacco, and tomato, were generated using the MEGA6 program by neighbor-joining analysis. Bootstrap values are displayed on the branches. The seven MtCAMTA proteins are boxed. The corresponding locus ID or NCBI GenBank accession numbers of *CAMTAs* from five dicot plants are shown in Table S2.

To investigate the phylogenetic relationships of *CAMTA* gene families, a phylogenetic tree of *CAMTAs* from five dicot plants including *M. truncatula*, *Arabidopsis*, soybean, tobacco, and tomato was constructed based on neighbor-joining (NJ) methods. Detailed information for these five *CAMTA* family genes is provided in Table S2. *CAMTA* gene families were highly conserved among these five dicot plants during the evolutionary process (Figure 3). All of the total 36 proteins from five dicot plants could be clustered distinctly into three groups (group A, B, and C). *MtCAMTA1*, *4*, and *7* were clustered into group A, together with *AtCAMTA1*, *2*, and *3*, which have been reported to play important roles in SA-mediated defense responses and cold tolerance together in *Arabidopsis* (Du et al., 2009; Kim et al., 2013a). In addition, six soybean *CAMTAs* (*GmCAMTA1*-*6*), three tomato *CAMTAs* (*SISR1*, *SISR1L*, and *SISR4*) and *NtER1* from tobacco belonged to group A. *MtCAMTA3* and *MtCAMTA6*, *AtCAMTA4*, *SISR2*, and *SISR2L*, and four soybean *CAMTAs* (*GmCAMTA10*, *11*, *14*, and *15*) fell into group B. *MtCAMTA2* and *MtCAMTA5*, *AtCAMTA5* and *AtCAMTA6*, *SISR3* and *SISR3L*, and four soybean *CAMTAs* (*GmCAMTA8*, *9*, *12*, and *13*) were clustered into group C.

### *Cis*-acting regulatory elements in the promoters OF *MtCAMTAs*

*Cis*-acting regulatory elements are specific motifs existing in the promoter regions of genes functioning as binding sites for transcription factors that regulate gene transcription (Liu et al., 2014). Identification and analysis of *cis*-acting regulatory elements present in the promoters of these genes help to expand our current understanding of the molecular regulation of these genes. We researched the −2000 bp upstream promoter regions of the seven *MtCAMTA* genes for several well-studied stresses/stimuli response *cis*-acting elements. Seven *cis*-elements were used in this study: ABA-responsive element (ABRE: C/TACGTGG/T) (Osakabe et al., 2014), SA-responsive promoter element (SARE: TGACG) (Pieterse and Van Loon, 2004), environmental signal response element (G-box: CACGTG) (Williams et al., 1992), WRKY binding site (W-box: TTGACC/T) (Chen et al., 2012), phosphate starvation-responsive element (P1BS: GNATATNC) (Rubio et al., 2001), sulfur-responsive element (SURE: GAGAC) (Maruyama-Nakashita et al., 2005), and the CAMTA binding site (CG-box) (Yang and Poovaiah, 2002; Choi et al., 2005).

The results showed that there were various known stresses/stimuli-related *cis*-acting elements that existed in the promoter regions of seven *MtCAMTAs* (Table 2). The upstream flanking regions of *MtCAMTA4* contained five types of *cis*-elements including ABRE, SARE, G-box, W-box, and CG-box. The upstream flanking region of *MtCAMTA1* and *MtCAMTA6* each had four types of stresses/stimuli-related *cis*-elements. *MtCAMTA1* contained ABRE, G-box, W-box, and SURE *cis*-elements in the promoter region, and the number of W-box reached five, indicating a possible transcriptional regulation by WRKY transcription factors. *MtCAMTA6* had SARE, G-box, CG-box, and P1BS *cis*-elements in the promoter regions. Three W-box, two CG-box and one P1BS were located on the promoter of *MtCAMTA7*, indicating transcriptional regulation by WRKY transcription factors and by the CAMTAs themselves. *Two* SARE and one CG-box were found in the promoter of *MtCAMTA2*. The promoter region of *MtCAMTA3* contained only one P1BS. Meanwhile, *MtCAMTA5* did not contain any chosen *cis*-element in its promoter region except for one CG-box.

**Table 2:** **Numbers of stress-related *cis*-elements in the upstream 2 kb regions of *MtCAMTA* genes**.

	**ABRE**	**SARE**	**G-box**	**W-box**	**CG-box**	**P1BS**	**SURE**
*MtCAMTA1*	1	0	1	5	0	0	2
*MtCAMTA2*	0	0	0	0	1	0	2
*MtCAMTA3*	0	0	0	0	0	1	0
*MtCAMTA4*	2	1	2	2	3	0	0
*MtCAMTA5*	0	0	0	0	1	0	0
*MtCAMTA6*	0	2	1	0	1	2	0
*MtCAMTA7*	0	0	0	3	2	1	0

*ABRE, ABA-responsive element; SARE, SA-responsive promoter element; G-box, environmental signal response element; W-box, WRKY binding site; P1BS, phosphate starvation-responsive element; SURE, sulfur-responsive element; CG-box, the CAMTA binding site*.

### Tissue-specific expression patterns of *MtCAMTA* genes

**Figure 4:**
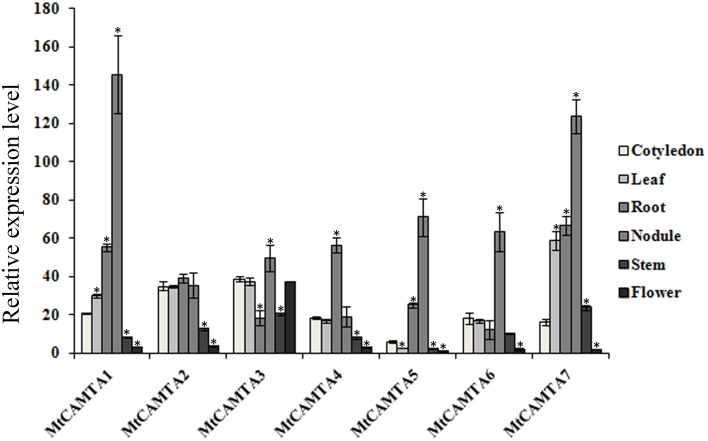
**Tissue-specific expression patterns of** ***MtCAMTA*** **genes**. Expression patterns of the *MtCAMTA* genes in five indicated tissues were analyzed by qRT-PCR. Relative expression was calculated based on the expression level of the target gene vs. the level of internal control gene *MtACTIN*, which was set to 1000. Each bar represents the mean of five biological replications with standard error. Asterisk denotes significance at *P* < 0.05 (Student's *t*-test) compared with the expression level of *MtCAMTA* genes in cotyledon.

To elucidate the possible functions of the *MtCAMTA* genes, their expression profiles were investigated by qRT-PCR in different tissues or organs, including the cotyledons from 1-week-old seedlings, leaves, roots, stems of 2-week-old seedlings, and the flowers from 2-month-old plants. As a result, transcripts of the seven *MtCAMTA* genes could be detected in all of the different tissues or organs, and their expression varied among tissues (Figure 4). The transcript levels of all the six *MtCAMTA* genes, except for *MtCAMTA3*, were highest in roots compared to other organs, while *MtCAMTA3* was mainly expressed in cotyledons, leaves, and flowers. Furthermore, RNA abundances of three *MtCAMTA* genes from group A (*MtCAMTA1*, *4*, and *7*) in roots were generally higher than genes from groups B and C in all other tissues.

Since the gene expression profiles for the majority of *M. truncatula* genes are available in the Medicago gene atlas (MtGEA, http://mtgea.noble.org/v3/), the expression data of *MtCAMTA* genes in different tissues were also searched and analyzed in the MtGEA (Benedito et al., 2008). The probeset ID of *MtCAMTA* genes has been listed in Table S3. Generally, these data showed the similar tissue-specific expression pattern for *MtCAMTA* genes (Figure S1).

### Expression profiles of *MtCAMTA* genes during the early phase of *Sinorhizobium meliloti* infection

Changes in intracellular Ca^2+^ signaling are well-documented features of legume-rhizobia interactions and nodule development (Lévy et al., 2004). During nodule formation, Ca^2+^ and calmodulin are critical players in plant responses to symbionts (Oldroyd and Downie, 2006; Ranty et al., 2006). In this study, we were interested in the putative roles of MtCAMTAs as calcium/calmodulin regulated transcription factors during nodule formation in *M. truncatula*, and we made our preliminary efforts to explore the involvement of MtCAMTAs during this process.

**Figure 5:**
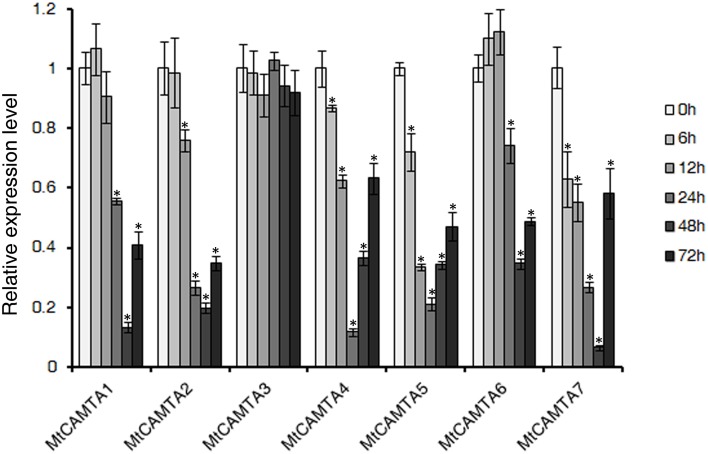
**Expression profile of MtCAMTA genes during the early phase of** ***S. meliloti*** **infection**. The expression levels of the *MtCAMTA* genes were analyzed by qRT-PCR at different time points (6/12/24/48/72 hpi) after *S. meliloti* infection. The expression levels of *MtCAMTA* genes in uninfected seedlings were normalized to a value of 1. Each bar represents the mean of five biological replications with standard error. Asterisk denotes significance at *P* < 0.05 (Student's *t*-test) compared with the expression levels of *MtCAMTA* genes in uninfected seedlings.

To investigate the expression responses of *MtCAMTA* genes during the early phase of *S. meliloti* infection, qRT-PCR was used to detect the expression of *MtCAMTA* genes under *S. meliloti* infection. Surprisingly, the expression levels of almost all the *MtCAMTA* genes showed a drastic decline during the early phase of *S. meliloti* infection in the roots, except for *MtCAMTA3*, which showed no detectable changes in the 72 h monitored period after *S. meliloti* infection (Figure 5). After 24 h infection, *MtCAMTA4* and *MtCAMTA5* reached a maximum repression of 5–10 folds. Meanwhile, *MtCAMTA1*, *2*, *6*, and *7* reached a maximum repression after 48 h infection. These results showed that the expression of six *MtCAMTAs* were responsive to early *S. meliloti* infection. The expression levels of *M. truncatula* early nodulin gene *MtENOD11*, used as a positive control, were also detected (Journet et al., 2001). The result showed that *MtENOD11* was strongly and rapidly induced during the early phase of *S. meliloti* infection (Figure S2).

In addition, the expression data of *MtCAMTA* genes under *S. meliloti* infection and nod factor treatment was searched and analyzed in the MtGEA (Czaja et al., 2012; Breakspear et al., 2014). The expression levels of *MtCAMTA* genes in root hairs showed no evident changes during the early phase of *S. meliloti* infection (Figure S3). Also, the expression levels of most *MtCAMTA* genes only slightly declined after 24 h nod factor treatment (Figure S3). As calcium spiking is also a key response to arbuscular mycorrhizal infection, the expression pattern of *MtCAMTA* genes under AM fungal signals Myc-LCOs treatment was also investigated in the MtGEA (Czaja et al., 2012). The expression levels of *MtCAMTA* genes showed no detectable changes under 6 and 24 h Myc-LCOs treatment in WT roots (Figure S3). However, the expression of all the *MtCAMTA* genes were suppressed after 6 and 24 h Myc-LCOs treatment in *dmi3* mutant roots (Figure S3).

### Analysis of the CAMTA binding sites in the promoter region of rhizobial infection response genes

Transcriptomics approaches have been used in *M. truncatula* and *Lotus japonicus* to obtain a view of the range of genes associated with early or late stages of nodulation (Fedorova et al., 2002; Colebatch et al., 2004; Lohar et al., 2006; Molesini et al., 2014). In *M. truncatula*, hundreds of plant genes involved in early stages of *S. meliloti* infection were identified using a microarray with about 6000 cDNAs (Lohar et al., 2006). We screened these genes and chose those with an expression ratio up to 2.5-fold. Then the CAMTA binding sites (A/C/G)CGCG(T/C/G) or (A/C)CGTGT DNA were analyzed in the promoter regions of these genes. Interestingly, about 45% of these early rhizobial infection response genes (73/162) contained CAMTA binding sites (Table S3), while the proportion for a random selection of genes with similar numbers was about 36%. These genes encode proteins involved in calcium transport and binding, reactive oxygen metabolism, cell proliferation, defense response and cytoskeleton and cell wall functions, and represent possible candidates for MtCAMTA-regulated downstream genes during the early phase of *S. meliloti* infection.

The CAMTA binding sites overlap with the ABA-responsive element, which is partly contained within in the CAMTA site (Kaplan et al., 2006). The ABA-responsive elements were also analyzed in the promoter regions of the infection regulated genes containing the CAMTA binding sites. A few of these genes contained the ABA-responsive elements in their promoter regions (Table S4). This result indicated that competition may exist between CAMTA binding and the ABA response for these infection regulated genes.

### Expression patterns of *MtCAMTA* genes in nodules

To investigate whether the *MtCAMTA* genes were expressed in nodules, qRT-PCR analysis was performed using total RNA from nodules. Transcripts of all the seven *MtCAMTA* genes could be detected in nodules, although the expression level of each gene varied in nodules (Figure 4). The expression levels of *MtCAMTA1* and *MtCAMTA7* in nodules were highest compared to other genes. *MtCAMTA4*, which was predominantly expressed in the roots, was rarely expressed in nodules.

Recently, laser-capture microdissection (LCM) has been successfully used in *M. truncatula* to precisely analyze gene expression in different zones of the nodules (Limpens et al., 2013; Roux et al., 2014). Specifically, expression data of *MtCAMTA* genes in different zones of the nodules were collected and analyzed based on the RNA sequencing coupling LCM resource (Table S5, Roux et al., 2014). In general, the seven *MtCAMTA* genes were expressed in all the five different zones of nodules, although the expression levels in each zone were different. *MtCAMTA2*, *6*, and *7* were predominantly expressed in the meristematic zone, while both *MtCAMTA2* and *6* were rarely expressed in the proximal infection zone. The expression of *MtCAMTA5* was enriched in the meristematic zone and the distal infection zone, while the expression was very low in the inter zone. *MtCAMTA1*, *3*, and *4* were equally expressed in the five zones of nodules, although slight differences existed. Data from Erik Limpens study also showed the similar results (Limpens et al., 2013).

### Expression of *MtCAMTA* genes in response to hormone treatments

**Figure 6:**
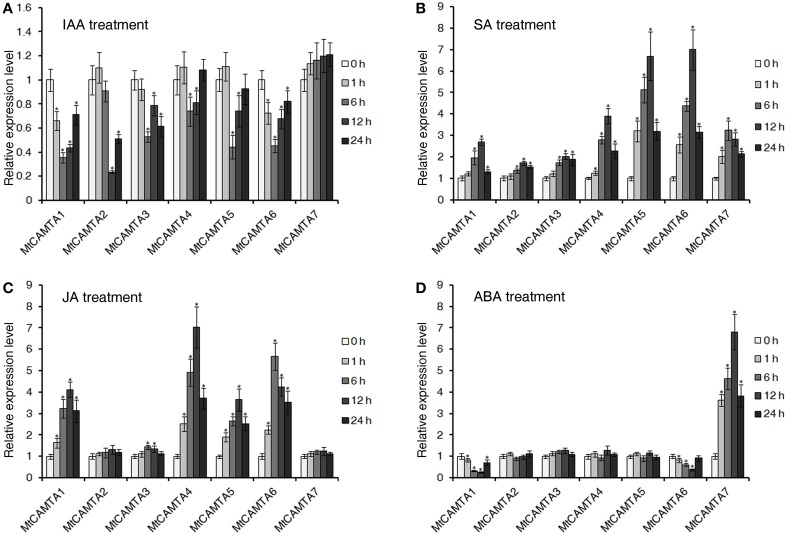
**Expression of** ***MtCAMTAs*** **in response to IAA, SA, JA, and ABA treatments**. Two-week-old *M. truncatula* seedlings were treated with 1 μM IAA **(A)**, 0.5 mM SA **(B)**, 100 μM Me-JA **(C)**, and 100 μM ABA **(D)**. The expression of seven *MtCAMTA* genes were analyzed by qRT-PCR in roots of hormone-treated and none-treated seedlings. The expression levels of *MtCAMTA* genes in none-treated seedlings were normalized to a value of 1. Each bar represents the mean of five biological replications with standard error. Asterisk denotes significance at *P* < 0.05 (Student's *t*-test) compared with the expression levels of *MtCAMTA* genes in none-treated seedlings.

It has been reported that *CAMTA* genes in *Arabidopsis* and soybean respond to various plant hormones, such as auxin, ABA, SA, and JA (Yang and Poovaiah, 2002; Galon et al., 2010b; Wang et al., 2014). Additionally, hormones such as auxin, SA, JA, and ABA act as positive or negative regulators of nodulation organogenesis and play central roles in coordinating plant responses to rhizobium infection (Suzuki et al., 2004; Stacey et al., 2006; Sun et al., 2006; Oldroyd and Downie, 2008). Here, the expressions of *MtCAMTA* genes in response to IAA, ABA, SA, and JA stresses were tested. As show in Figure 6A, all the *MtCAMTA* genes were reduced in the root under IAA treatment, except for *MtCAMTA7*, which showed no detectable changes. For SA treatment, all the *MtCAMTA* genes were induced quickly after 1 h treatment, and reached a peak after 6 or 12 h treatment (Figure 6B). The expression of *MtCAMTA5* and *6* showed a dramatic increase after SA treatment, while *MtCAMTA3* was slightly induced by SA treatment (Figure 6B). Under JA treatment, the expression of *MtCAMTA1*, *4*, *5*, and *6* were considerably induced, and reached a maximum fold after 6 to 12 h treatment (Figure 6C). For ABA treatment, only *MtCAMTA7* was induced, and the expression level reached the maximum under 12 h treatment; while the expression of *MtCAMTA1* and *6* were suppressed by ABA treatment (Figure 6D). The results suggested that the expression of *MtCAMTA* genes responded to the four nodulation-regulated and stress-related hormones, including IAA, ABA, SA, and JA.

Considering the close relationship of SA, JA, and pathogens, it is interesting to investigate whether *MtCAMTA* genes were responsive to pathogens infection. Here, a preliminary investigation was undertaken to gain some insight. The expression data of *MtCAMTA* family genes during infection with the three pathogens in the MtGEA database were analyzed. The result showed that *MtCAMTA* genes were differentially responsive to *Ralstonia solanacearum* and Phymatotrichum Root Rot infection, while no detectable expression changes occurred in response to *Macrophomina phaseolina* infection (Figure S4).

## Discussion

CAMTA transcription factors are important players in the calcium/calmodulin transduction signal pathway. So far, the features and functions of *CAMTA* family genes have already been investigated and identified in several plant species, including *Arabidopsis*, tomato, tobacco, soybean, and rice (Yang and Poovaiah, 2002; Du et al., 2009; Koo et al., 2009; Yang et al., 2012; Wang et al., 2014). However, to date there are still no studies on *CAMTAs* in *M. truncatula*, an important leguminous model plant that is widely used in symbiosis research. Genome-wide analysis of the *CAMTA* genes in *M. truncatula* would facilitate a better understanding of the role of this gene family during nodule formation.

### Characterization and expression patterns of the *MtCAMTA* gene family in *Medicago truncatula*

In this study, seven members of *M. truncatula CAMTA* family genes were identified. The number of *M. truncatula CAMTA* genes was similar to *Arabidopsis* (six members) and much smaller than soybean (15 members), although the *Medicago* genome size is approximately three times that of the *Arabidopsis* genome and half of the soybean genome (Young et al., 2011). The higher number of soybean *CAMTA* genes may be explained by the allotetraploid nature of soybean.

Similar to all of the CAMTAs that have been characterized in various species, all of the seven family members contain conserved domains of CAMTAs (Figure 2). A combined N-J tree was also constructed to investigate the phylogenetic relationships of *CAMTA* genes in plants and their evolutionary relationships. Based on the phylogenetic tree, we found a close relationship exists among *CAMTAs* in five dicot plants, suggesting that the functions of *MtCAMTAs* could be similar to *CAMTAs* in other plant species (Figure 3). Interestingly, three members of the *CAMTAs* gene family (*MtCAMTA1*, *4*, and *7*) showed a close relationship with three *Arabidopsis CAMTA* genes (*AtCAMTA1*, *2*, and *3*) that have been well-studied and are known to participate together in SA-mediated defense responses and cold tolerance (Doherty et al., 2009; Kim et al., 2013b). These results indicate that *MtCAMTA1*, *4*, and *7* have a close relationship with each other and may function together in the same pathway as homolog genes. The expression patterns of *CAMTAs* in different groups from three different species (including *Arabidopsis*, *M. truncatula*, and soybean) were compared under hormones treatment. Most of the *CAMTA* members from group A were induced by SA, JA, and ABA treatment. For group B, all the *CAMTAs* in this group were induced by SA; most of them were induced by JA; while most of them were not induced and even repressed under ABA treatment. Most of the *CAMTA* members from group C were induced by SA and JA; for ABA treatment, the expression of *AtCAMTAs* and *GmCAMTAs* were up-regulated, while there was no detectable expression changes for the two *MtCAMTAs*. This results indicated that *CAMTAs* from each group may have distinctive expression patterns under hormones treatment.

The tissue-specific expression analysis of *MtCAMTA* genes showed differential expression in various *M. truncatula* tissues and organs (Figure 4). The different expression patterns of *MtCAMTA* genes suggested that they may play different roles in plant growth and development. In particular, six genes are mainly expressed in the root. This root-preferential expression type indicated a possible role of *MtCAMTA* genes in response to environmental stimuli and bacterial infection in soil. The expression levels of *MtCAMTA1*, *4*, and *7* in roots were higher than the other four genes in any tissue, emphasizing their important role in root growth in *M. truncatula*.

As signal response genes, *CAMTAs* were reported to be responsive to diverse stresses and stimuli in *Arabidopsis*, tomato, and soybean (Yang and Poovaiah, 2000; Galon et al., 2010b; Yang et al., 2012; Wang et al., 2014). Enrichment of *cis*-elements involved in stresses/stimuli response in *MtCAMTA* promoters suggest that they are likely to respond differently to various stresses and stimuli signals, like other *CAMTAs* in different species. The enrichment of the CG-box in the promoter regions of most *MtCAMTA* family members suggested comprehensive transcriptional regulation by the CAMTAs themselves, and indicated a complicated regulation network between them.

### The putative function of MtCAMTAs in nodule formation during the early phase of *Sinorhizobium meliloti infection*

During nodule formation, calcium plays an essential role as a secondary messenger. It is convinced that CCaMK is a key calcium sensor in the early nodulation signaling pathway (Mitra et al., 2004). After binding to Ca^2+^ or Ca^2+^/calmodulin, CCaMK induces the epidermal expression of specific early nodulation genes via the phosphorylation of transcription factors like CYCLOPS (Lévy et al., 2004; Mitra et al., 2004; Yano et al., 2008; Singh and Parniske, 2012; Singh et al., 2014). Apart from CCaMK, several CaM, CML genes and calcium/calmodulin binding proteins were also found to be expressed in *Medicago* and *Lotus* nodules by expression profile analysis (Fedorova et al., 2002; Colebatch et al., 2004; Moreau et al., 2011). However, with the exception of CCaMK, a well-known important regulator of plant-microbe symbioses, the role of Ca^2+^ in fully developed nodules was less clear. The involvement of other players in the calcium signal transduction pathway of symbiosis is still largely unknown and needs further research. Our results showed that the expression levels of *MtCAMTA* genes responded to *S. meliloti* infection in *M. truncatula*. The expression of all but one *MtCAMTA* genes were dramatically suppressed during *S. meliloti* infection (Figure 5). This expression pattern indicated that MtCAMTAs may be involved in the transduction of the early nodulation signal as calcium regulated transcription factor in symbiosis. To date, several important transcription factors have been found to play important roles in symbiosis, including CYCLOPS, NIN, NF-Y, NSPs, SIP1, and IPN2, which was reported most recently (Soyano and Hayashi, 2014). However, there is no report about the involvement of CaM-binding transcription factors in symbiosis. This study showed that the *MtCAMTA* gene family may be a novel CaM-binding transcription factor family involved in the early phase of infection.

Previous work has shown that CAMTAs can act as either positive or negative regulators after binding to calcium/calmodulin (Du et al., 2009; Nie et al., 2012; Kim et al., 2013b). Based on *cis*-elements analysis, we found that most of the nodulation genes that are up-regulated or down-regulated during the early phase of *S. meliloti* infection contained the CAMTA binding site in their promoter regions (Table S4). This result indicated that MtCAMTAs can possibly bind to the nodulation genes and regulate their expression as transcription factors. Therefore, it can be inferred that calcium can regulate the nodulation signaling pathway by forming a calcium/calmodulin complex to activate MtCAMTAs, which then modulate the expressions of downstream genes associated in the nodulation signaling pathway positively or negatively as transcription factors. Considering the early down-regulation of *CAMTA* genes during rhizobial infection, it can be inferred that these factors need to be down-regulated to allow infection and they may be negative regulators of infection. However, this presumption still needs to be confirmed, and the candidate downstream regulated genes should be screened by further investigation.

During the early stages of *M. truncatula* infection, the morphology of the root hairs changed, including swelling of root hair tips by 1 hpi, the asymmetrical root hair tip by 6 hpi, hair branching by 12 hpi, and curled root hairs by 24 hpi (Lohar et al., 2006). Inner cortical cells started dividing between 24 and 48 hpi, and the infection threads initiated in the tightly curled root hairs by 48 hpi (Lohar et al., 2006). At 72 hpi, infection threads penetrated the cortical cells and nodule primordia were observed (Lohar et al., 2006). During the early phase of infection, the expression levels of all the *MtCAMTA* genes, with the exception of *MtCAMTA3*, showed a drastic decline, and reached a maximum fold of repression after 24 or 48 h infection (Figure 5). In addition, microarray data from the MtGEA also showed that no evident changes occurred for *MtCAMTA* genes in root hairs during the early phase of *S. meliloti* infection (Figure S3). Thus, it can be inferred that the down-regulation of *MtCAMTA* genes may be needed mainly in the phase of inner cortical cells dividing. However, further investigation should be undertaken to confirm this presumption.

Our results showed that all the seven *MtCAMTAs* were expressed in nodules, although the expression level of each gene varied in nodules (Figure 4). This result indicates that the seven *MtCAMTA* genes may be involved in nodule morphogenesis. Based on the RNA sequencing coupling LCM resource, we also found that the expression levels of *MtCAMTAs* in each zone of the nodules were different, indicating that they may play different roles in the different stages of nodule morphogenesis. Furthermore, we took advantage of the microarray data (GPL4592) to investigate the expression pattern of *CAMTA* gene in another legume model plant (soybean). The data showed that the expression of most *CAMTA* genes is down-regulated in the roots with nodules compared to the roots used as control (Figure S5, Table S6). It suggested that the down-regulation of *CAMTA* genes may be conserved in legume.

### *MtCAMTA* genes were responsive to hormones which had critical roles in the regulation of nodule organogenesis

The involvement of *CAMTA* genes in response to plant hormones has been well-studied in *Arabidopsis* and soybean (Yang and Poovaiah, 2002; Galon et al., 2010b; Wang et al., 2014). Previous studies indicated that four *Arabidopsis CAMTA* genes were quickly induced by ABA and SA treatment (Yang and Poovaiah, 2002). *AtCAMTA1* was induced by endogenous auxin, and has a possible role in auxin signaling (Galon et al., 2010b). Recently, 15 soybean *CAMTA* genes were identified, and their expression was responsive to three major stress-related hormones, including ABA, SA, and JA (Wang et al., 2014). Our investigation on the response of *MtCAMTAs* to four major stress-related hormones came to a similar conclusion. The expressions of most *MtCAMTA* genes were responsive to the four hormones, including IAA, SA, JA, and ABA (Figure 6).

Plant hormones play critical roles in the regulation of nodule organogenesis in legumes (Hirsch and Fang, 1994; Oldroyd and Downie, 2008). Auxin was reported to act as positive regulator in nodulation initiation in *M. truncatula* roots (Oldroyd and Downie, 2008). Inhibition of auxin polar transport at the nodule site could precede the earliest stages of root nodule formation in legumes (Mathesius et al., 1998). High levels of endogenous auxin have been correlated with increased numbers of nodules, while reduction of auxin levels in the root inhibits further nodule initiation in *M. truncatula* (van Noorden et al., 2006). Our data showed that the expression of almost all of the *MtCAMTA* family members was repressed by auxin treatment (Figure 6A). Interestingly, this result correlated with the expression inhibition of *MtCAMTA* genes by *S. meliloti* treatment (Figure 5).

In contrast to auxin, three major stress-related hormones SA, JA, and ABA all act as negative regulators to repress nodulation (Hirsch and Fang, 1994; van Noorden et al., 2006). Reduction of endogenous SA levels elevated the number of infections and the mean nodule number in both *L. japonicus* and *M. truncatula* plants (Stacey et al., 2006). JA suppressed the early stages of nodulation by inhibiting Nod factor-induced calcium spiking and calcium oscillations (Nakagawa and Kawaguchi, 2006; Sun et al., 2006). Exogenous ABA blocked the step between root hair swelling and curling during nodule initiation, and inhibited root nodule formation after inoculation with rhizobia (Suzuki et al., 2004). Our investigation showed that while under these three nodulation-negative-regulated hormones treatment, most of the *MtCAMTA* family members were induced (Figures 6B–D). The expression of most *MtCAMTA* genes was significantly induced (greater than four-fold), with SA and JA treatments. These results may indicate a complicated relationship between hormone regulation and expression of *MtCAMTA* family members in nodule formation. Under hormones treatment, most of *MtCAMTAs* were induced or repressed, and reached the maximum level after 6 or 12 h treatment (Figure 6). Compared to the repression of *CAMTAs* during infection, their expressions reached the maximum fold earlier under hormones treatment. It can be assumed that auxin inhibits the expression of *MtCAMTA* genes during the early phase of infection, thus promoting the process of nodulation. whereas SA, JA, and ABA may induce *MtCAMTA* genes to repress nodulation.

*M. truncatula* is an important leguminous model plant that is widely used in studies of symbiotic association with N-fixing bacteria. The present study has identified and characterized all of the *CAMTA* genes in *M. truncatula*. The identification, chromosomal location, protein domain, and expression profiling analysis of *MtCAMTA* genes in different tissues were all investigated in detail. Expression profile analysis of *MtCAMTAs* during early nodulation process indicated that CAMTAs in *M. truncatula* may be important transcription factors during the early phase of *S. meliloti* infection. These results should provide a solid foundation for future functional studies and also in guiding subsequent experimental work on *CAMTA* genes in this model species.

### Conflict of interest statement

The authors declare that the research was conducted in the absence of any commercial or financial relationships that could be construed as a potential conflict of interest.
