# Supplementary material for: Genome-wide identification of CAMTA gene family members in Medicago truncatula and their expression during root nodule symbiosis and hormone treatments
Source: Front Plant Sci. 2015 Jun 19;6:459. doi: 10.3389/fpls.2015.00459 (PMC4472986; doi:10.3389/fpls.2015.00459)
Supplement: Supplementary file 7 [file Image1.PDF]

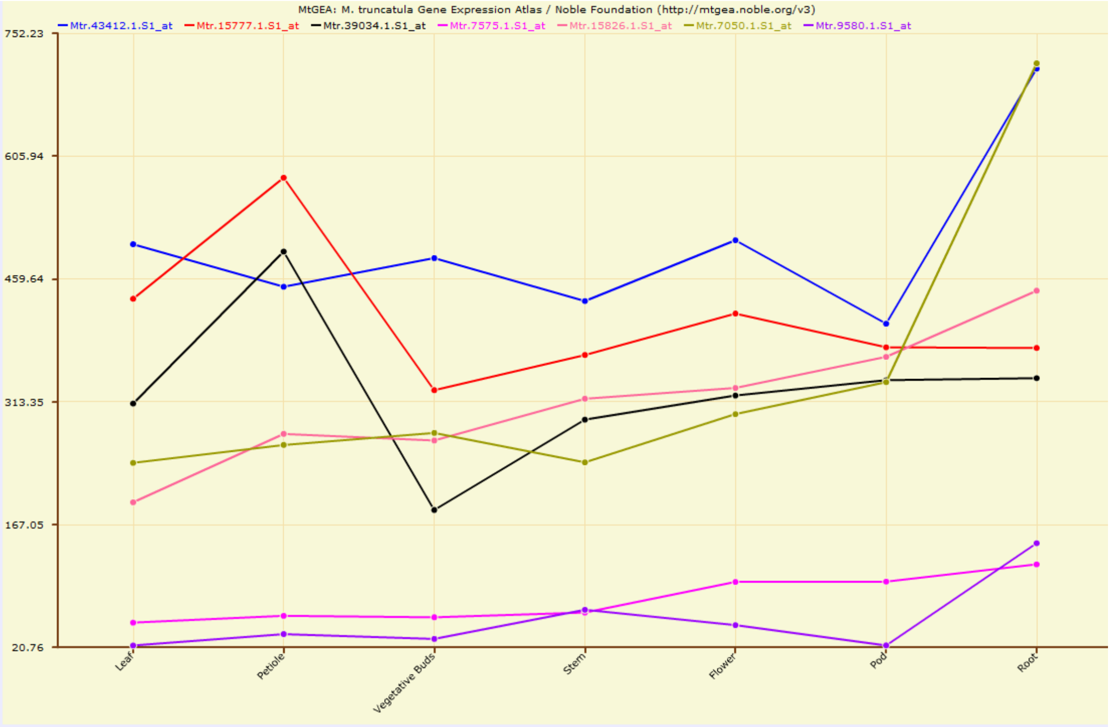

Figure S1 The tissue-specific expression pattern of *MtCAMTA* family genes from *Medicago* gene atlas (http://mtgea.noble.org/v3/). The probeset ID for each *MtCAMTA* gene was listed in the Table S3.
