# Supplementary material for: Genome-wide identification of CAMTA gene family members in Medicago truncatula and their expression during root nodule symbiosis and hormone treatments
Source: Front Plant Sci. 2015 Jun 19;6:459. doi: 10.3389/fpls.2015.00459 (PMC4472986; doi:10.3389/fpls.2015.00459)
Supplement: Supplementary file 8 [file Image2.PDF]

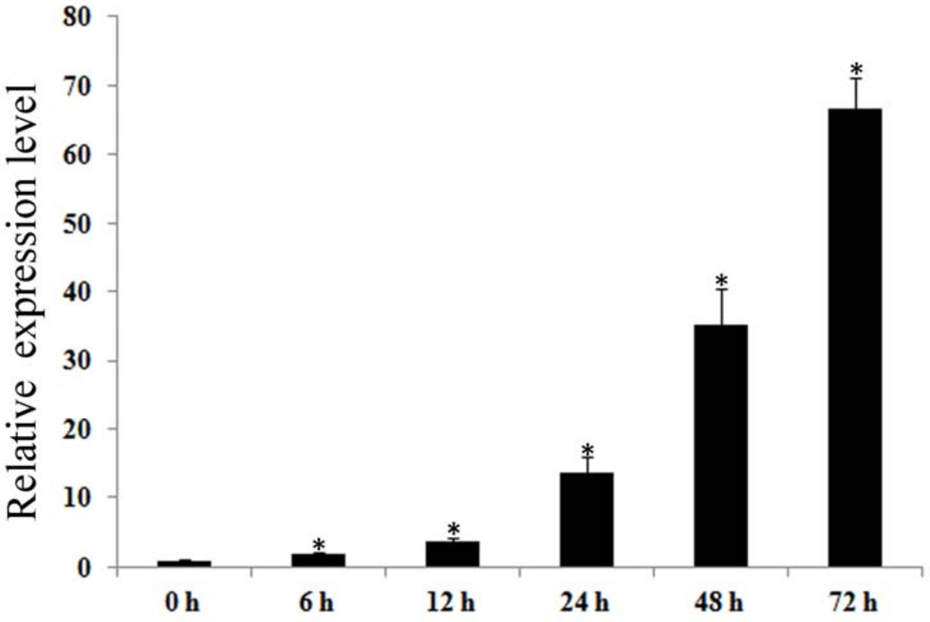

Figure S2 Expression profile of *MtENOD11* genes as a positive control during the early phase of *S. meliloti* infection. The expression levels of the *MtCAMTA* genes were analyzed by qRT-PCR at different time points (6 /12 /24 /48 /72 hpi) after *S. meliloti* infection. The expression levels of *MtENOD11* genes in uninfected seedlings were normalized to a value of 1. Each bar represents the mean of five biological replications with standard error. Asterisks denotes significance at $P<0.05$ (Student's *t* test) compared with the expression levels of *MtCAMTA* genes in uninfected seedlings.
