# Supplementary material for: Genome-wide identification of CAMTA gene family members in Medicago truncatula and their expression during root nodule symbiosis and hormone treatments
Source: Front Plant Sci. 2015 Jun 19;6:459. doi: 10.3389/fpls.2015.00459 (PMC4472986; doi:10.3389/fpls.2015.00459)
Supplement: Supplementary file 9 [file Image3.PDF]

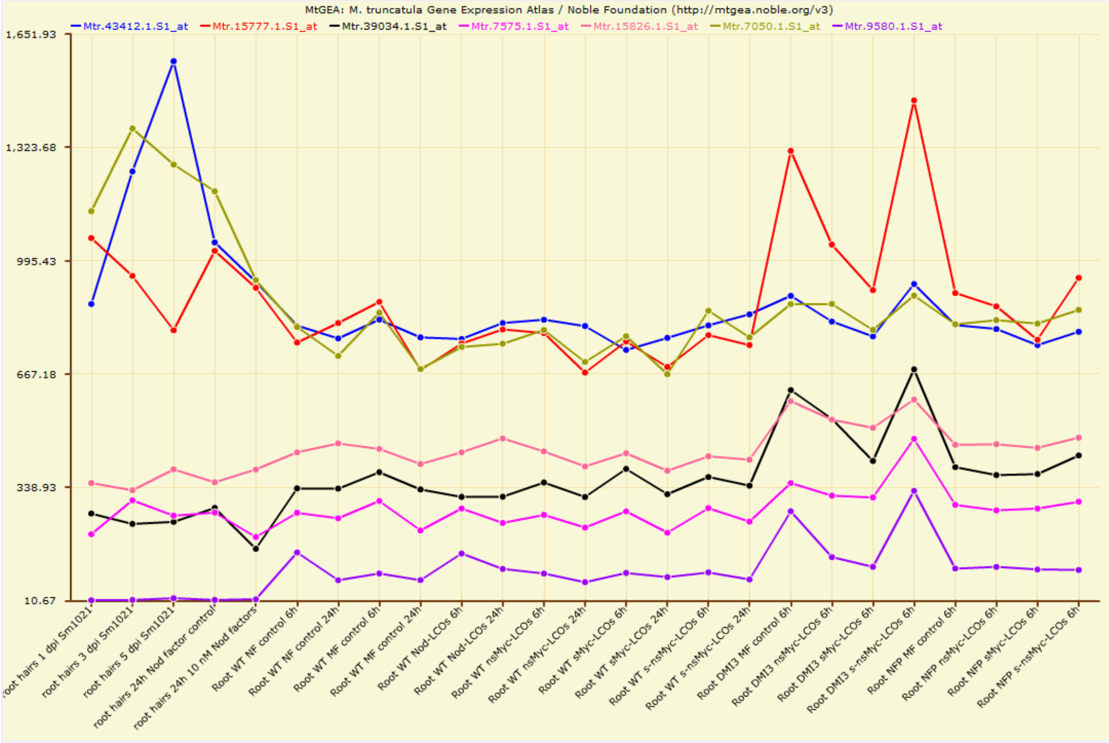

Figure S3 Expression data of *MtCAMTA* genes under *S. meliloti* infection, nod factor treatment and Myc-LCOs treatment from *Medicago* gene atlas (http://mtgea.noble.org/v3/). The expression levels of the *MtCAMTA* genes in root hairs were analyzed at different time points (1 /3 /5 dpi) after *S. meliloti* infection and 6 /24 h nod-LCOS and Myc-LCOs treatment.
