# Supplementary material for: Genome-wide identification of CAMTA gene family members in Medicago truncatula and their expression during root nodule symbiosis and hormone treatments
Source: Front Plant Sci. 2015 Jun 19;6:459. doi: 10.3389/fpls.2015.00459 (PMC4472986; doi:10.3389/fpls.2015.00459)
Supplement: Supplementary file 10 [file Image4.PDF]

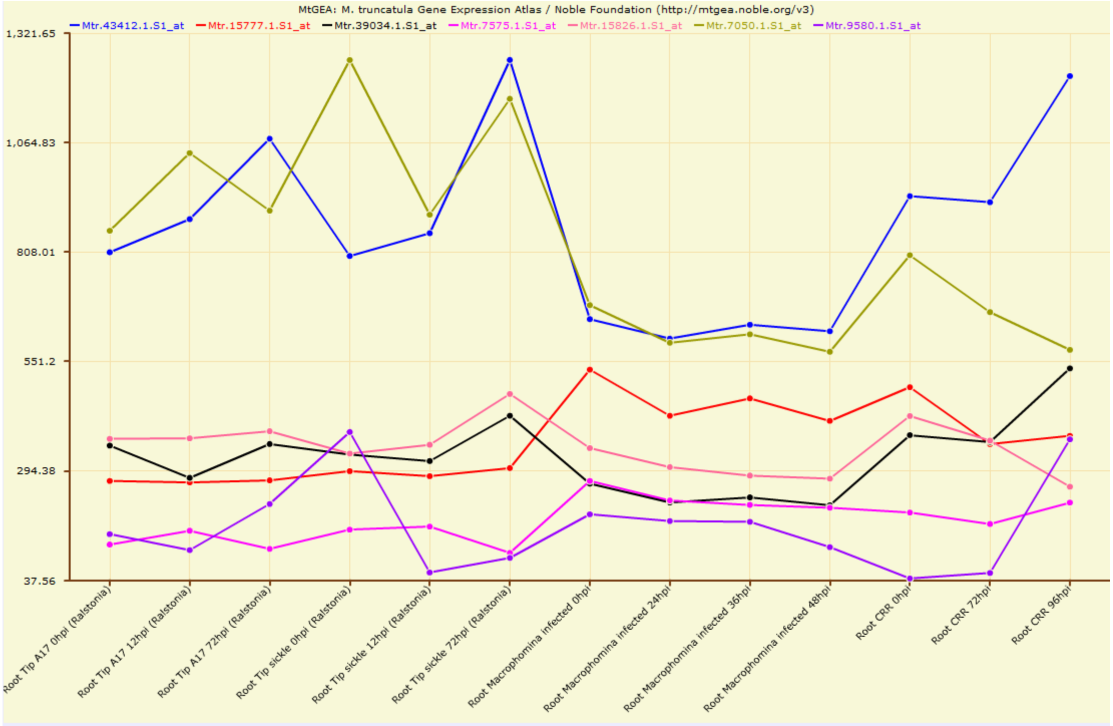

Figure S4 Expression data of *MtCAMTA* genes during pathogens infection from *Medicago* gene atlas (http://mtgea.noble.org/v3/). Ralstonia: *Ralstonia solanacearum*; Macrophomina: *Macrophomina phaseolina*; CRR: Phymatotrichum Root Rot.
