# Supplementary material for: Genome-wide identification of CAMTA gene family members in Medicago truncatula and their expression during root nodule symbiosis and hormone treatments
Source: Front Plant Sci. 2015 Jun 19;6:459. doi: 10.3389/fpls.2015.00459 (PMC4472986; doi:10.3389/fpls.2015.00459)
Supplement: Supplementary file 11 [file Image5.PDF]

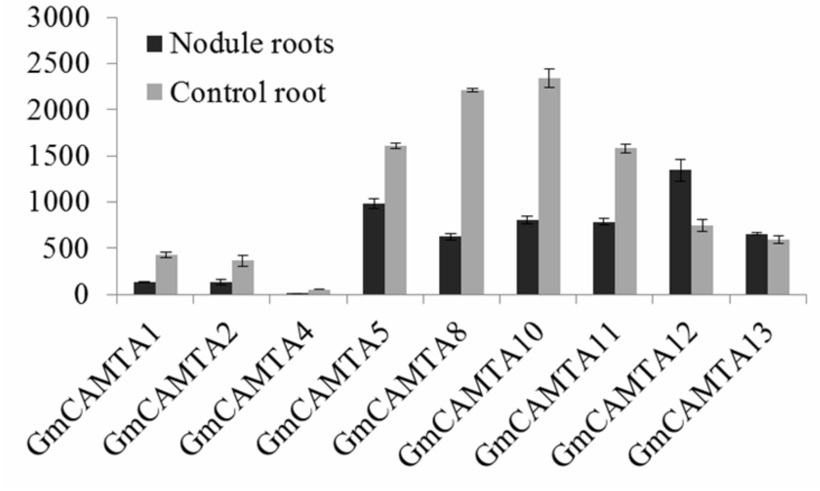

Figure S5 The expression pattern of *CAMTA* genes in soybean. The expression value was obtained from the microarray data (GPL4592). The probeset ID for each *GmCAMTA* gene was listed in the Table S6.
